# GPA-14, a Gα_i_ subunit mediates dopaminergic behavioral plasticity in *C. elegans*

**DOI:** 10.1186/1744-9081-9-16

**Published:** 2013-04-22

**Authors:** Mahlet Mersha, Rosaria Formisano, Rochelle McDonald, Pratima Pandey, Nektarios Tavernarakis, Singh Harbinder

**Affiliations:** 1 Department of Biological Sciences, Delaware State University, Dover, DE 19901, USA; 2 Institute of Molecular Biology and Biotechnology, Foundation for Research and Technology-Hellas, Medical School, University of Crete, Heraklion, Crete 70013, Greece

**Keywords:** *C. elegans*, Learning, Habituation, Memory, *gpa-14*, *dop-2*, Dopamine, Dopamine receptor, G-protein, G*α*

## Abstract

**Background:**

Precise levels of specific neurotransmitters are required for appropriate neuronal functioning. The neurotransmitter dopamine is implicated in modulating behaviors, such as cognition, reward and memory. In the nematode *Caenorhabditis elegans*, the release of dopamine during behavioral plasticity is in part modulated through an acid-sensing ion channel expressed in its eight dopaminergic neurons. A D2-like *C. elegans* dopamine receptor DOP-2 co-expresses along with a Gα_i_ subunit (GPA-14) in the anterior deirid (ADE) pair of dopaminergic neurons.

**Findings:**

In follow-up experiments to our recently reported *in vitro* physical interaction between DOP-2 and GPA-14, we have behaviorally characterized worms carrying deletion mutations in *gpa-14* and/or *dop-2*. We found both mutants to display behavioral abnormalities in habituation as well as associative learning, and exogenous supply of dopamine was able to revert the observed behavioral deficits. The behavioral phenotypes of *dop-2* and *gpa-14* loss-of-function mutants were found to be remarkably similar, and we did not observe any cumulative defects in their double mutants.

**Conclusion:**

Our results provide genetic and phenotypic support to our earlier *in vitro* results where we had shown that the DOP-2 dopamine receptor and the GPA-14 Gα_i_ subunit physically interact with each other. Results from behavioral experiments presented here together with our previous *in-vitro* work suggests that the DOP-2 functions as a dopamine auto-receptor to modulate two types of learning, anterior touch habituation and chemosensory associative conditioning, through a G-protein complex that comprises GPA-14 as its Gα subunit.

## Findings

Neural plasticity is dependent upon various neurotransmitters, including the catecholamine dopamine [1,2] and abnormal dopaminergic transmission is associated with memory disorders [3]. Dopamine receptive neurons have dopamine receptors mainly localized to the morphologically plastic dendritic spine regions [4]. In humans, these seven-transmembrane G-protein coupled dopamine receptors are classified into D1- and D2-types. Activated D1-type receptors couple to Gα_s_ and activate adenylyl cyclase, and D2-receptors tend to act antagonistically to D1-receptors, mediating the signal transduction through Gα_i_[5]. Dopamine released into the synaptic cleft by presynaptic neurons interacts with its receptors after which it is either degraded by monoamine oxidase or taken up through a dopamine transporter [6]. The release of dopamine from acidic vesicles is accompanied by increase in H^+^ ion concentrations stimulating presynaptic acid-sensing ion channels (ASICs) that are proposed to modulate levels of dopamine in the synaptic cleft [7,8]. Some mammalian D2-receptors are localized to dopamine releasing neurons in a pre-synaptic configuration and thereby act as auto-receptors [9].

*Caenorhabditis elegans* is an ideal invertebrate model to study genes involved in behavioral plasticity [10-12]. In the adult hermaphrodite *C. elegans*, dopamine is synthesized in eight neurons: two anterior deirid neurons (ADEs), two posterior deirid neurons (PDEs) and four cephalic neurons (CEPs) [13]. Four dopamine receptor genes have been identified in the *C. elegans* genome: *dop-1, dop-2, dop-3* and *dop-4*. Based on pharmacological properties of their protein products and their sequence profiles, DOP-1 is classified as a D1-type receptor, while DOP-2 and DOP-3 are classified as D2-type receptors and DOP-4 is invertebrate specific [14]. Loss-of-function mutants for *dop-1* tend to habituate faster [15,16] and loss-of-function *dop-2* mutants display associative learning deficits [8]. DOP-3 and DOP-4 have both been implicated in response to aversive soluble repellents [17,18]. Seven-transmembrane receptors such as the dopamine receptors typically transduce their signal through G-proteins. The *C. elegans* genome encodes for 21 Gα, 2 Gβ and 2 Gγ genes; one particular gene, *gpa-14,* codes for a Gα_i_ subunit and shows expression overlap with DOP-2 as well as with ASIC-1 in the ADE dopaminergic neurons [8,19,20]. We have recently reported physical interaction between DOP-2 and GPA-14 and that the third intracellular loop of DOP-2 is essential for binding to GPA-14 *in vitro*[21]. To study the functional significance of the above molecular interaction in the intact organism, results from follow-up behavioral and genetic experiments are presented here.

## Hypothesis

Considering the role of dopamine in plasticity and the interaction of DOP-2 with GPA-14, we hypothesized that deletion of *gpa-14* will cause behavioral abnormalities similar to *dop-2* mutants [8,21]. Towards this end, we report that both *dop-2(vs105)* and *gpa-14(pk347)* loss-of-function mutants display associative learning deficits as well as faster habituation at remarkably similar rates. Additionally, the phenotype of the *gpa-14(pk347);dop-2(vs105)* double mutant is virtually identical to either single-mutant, and exogenous dopamine tends to revert the mutant behavior.

### *gpa-14* and *dop-2* mutants display similar behavioral deficits

**Figure 1:**
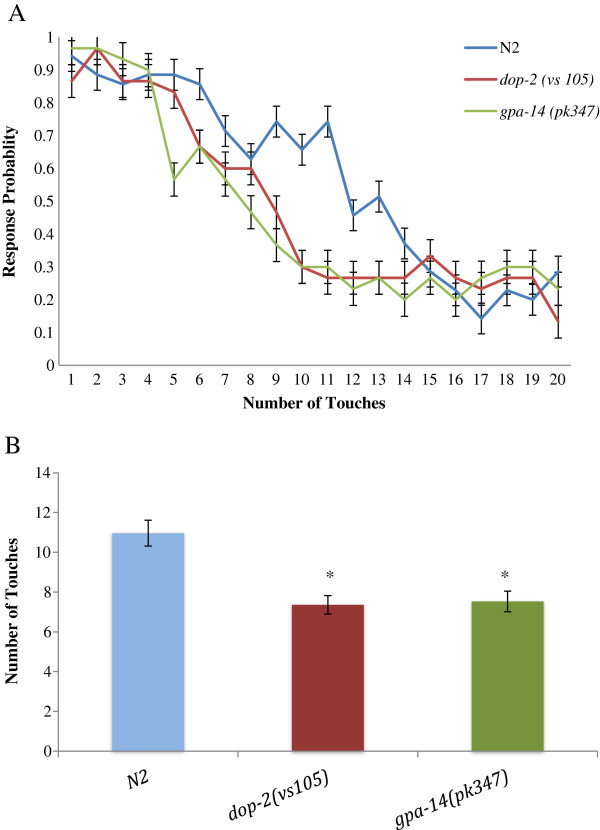
**Habituation measure based on response failure after repeated gentle anterior touch.** (**A**) 20 gentle anterior touches each separated by 10 second inter-stimulus interval were given and the response for each touch was scored as 1 if thw worm responded by moving away, and 0 if there was no response or worm continued moving in same direction. The probability of the worms responding to the touch decreased with increasing number of stimuli. (**B**) The raw data used in Figure-1A was plotted as a bar graph to illustrate that the average habituation point of *dop-2(vs105)* and *gpa-14(pk347)* deletion mutants was significantly lower than WT animals (p < 0.05, two tailed student’s t-test, n = 30 for each strain). Error bars represent SEM values.

**Figure 2:**
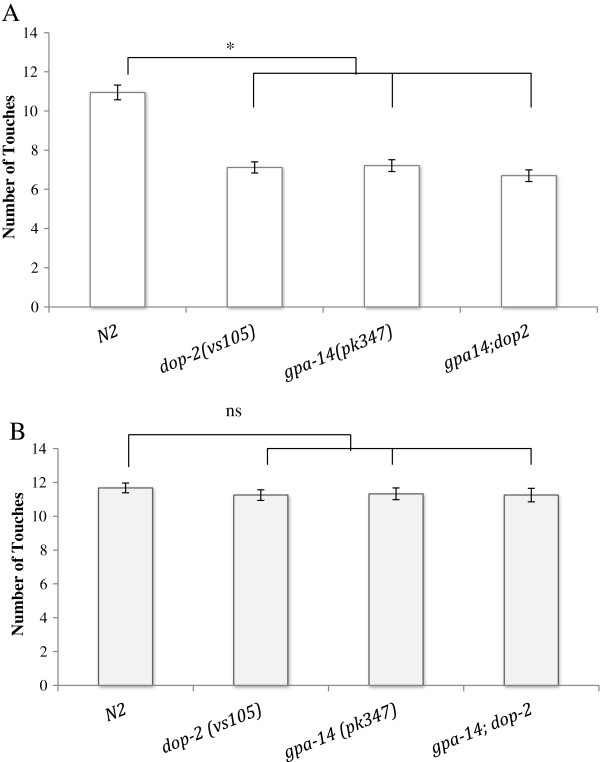
**Deletion mutants in *gpa-14 *and *dop-2 *habituate faster than WT, and are lowered to WT levels by exogenous dopamine.** Nematode Growth Media (NGM) plates were prepared fresh the night before the assay and left overnight at room temperature. Before the assay, 10 young adult worms were transferred to the NGM plates without food. Worms were gently touched on the anterior with an eyelash hair every 10 seconds until habituated. In response to this stimulus, the worms typically move backwards. The number of times the animal moved backward until it no longer responds to the stimulus was counted. The experiment was repeated by adding 60 μl of 5 mM dopamine spread on the 90 mm plates and allowed to dry for 10 minutes. (**A**) Both *gpa-14(pk347)* and *dop-2(vs105)* mutants habituate faster than wild type (*N2*) animals. (p < 0.05, Student’s two-tailed t-test, n = 55). (**B**) After the application of exogenous dopamine, the habituation rates of the mutants reverted back to the wild type rates. (p = 0.37 Student’s two-tailed t-test, n = 40; error bars represent SEM values). The habituation rate of the *gpa-14(pk347);dop-2(vs105)* double mutant is not significantly different to either single mutant when tested in the absence (p = 0.18, Student’s two-tailed T-test) or presence of dopamine (p = 0.43, Student’s two-tailed t-test).

We carried out a behavioral profiles for *gpa-14(pk347)* and *dop-2(vs105)* mutants obtained through the *Caenorhabditis* Genetic Center, and cultured on standard nematode growth media *with E. coli* OP50 at 20°C. Compared to wild type N2 animals, individuals of both strains are phenotypically normal in terms of body size, shape, growth, movement and locomotion and they respond normally to gentle touch, and display normal chemotaxis to both soluble and volatile chemicals. Dopamine has been reported to mediate movement and food sensing behaviors in that when *C. elegans* encounter food, they move more slowly [2]. Our basal slowing assays did not reveal any significant difference between wild type animals and the deletion mutants (Additional file 1). A modified habituation assay was used in which a forward moving worm was gently touched on the anterior with an eyelash hair causing the worm to move backwards [22]. The mechanical stimulus was repeated with 10 sec intervals until the animal no longer responded to the stimulus. In order to confirm that the novel anterior touch based assay used here was in fact measuring habituation, we initially provided 20 gentle anterior touches (with 10 sec inter-stimulus intervals) to each worm and their response was scored after each stimulus as 1 or 0 (1 = worm moves away, and 0 = no response or worm continues moving in same direction). We observed that increasing number of touches decreased the probability of the worms’ response and we did not observe abrupt disruption in their ability to respond (Figure 1A). The latter would have indicated sensory fatigue. We also plotted the same data in terms of the average point at which worms stopped responding to the anterior touch stimulus (Figure 1B). Based on assay similarities and substantial neural circuitry overlap of the observed behavior with the extensively studied mechanical tap habituation, we consider that a decrease in response to anterior touch is a form of habituation, although additional tests will allow necessary verification [22,23]. Our subsequent assays recorded the number of mechanical stimuli repeated with 10 second intervals until the response failed as a measure of habituation of individual worms. The results are noteable in that both *dop-2(vs105)* and *gpa-14(pk247)* mutants exhibited significantly faster habituation rates compared to wild-type (Figures 1A and B, 2A).

**Figure 3:**
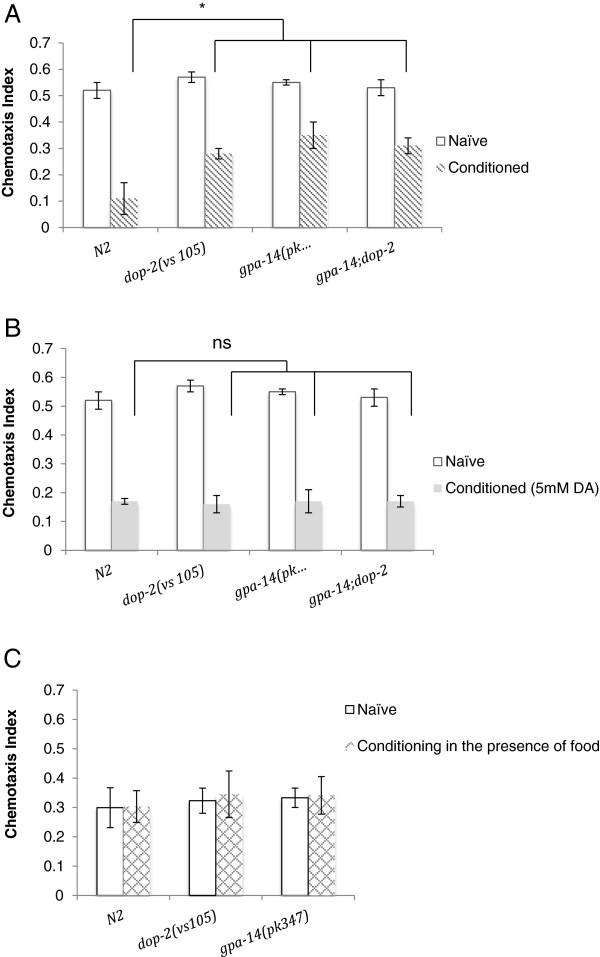
**Deletion mutants in *gpa-14 *and *dop-2 *display limited associative learning that is rescued by exogenous dopamine.** Associative learning chemotaxis assay. Three-day-old synchronized worms were conditioned with 3 μl of isoamyl alcohol on freshly prepared chemotaxis plates and tested with 1:100 isoamyl alcohol in the absence of food, as described previously [8,24]. Plates were left undisturbed for one hour and then kept at −10°C for 3 minutes. Chemotaxis index was calculated by subtracting the number of worms at the diluent sector from the number of worms at the chemical gradient sector and dividing by the total number of worms on the plate. Tests were then repeated in the presence of plates supplemented with 60 μl of 5 mM exogenous dopamine. (**A**) While naïve strains [wild type, *dop-*2*(vs105)* and *gpa-14(pk347)*] are equally attracted to isoamyl alcohol but after conditioning, wild type worms display significantly greater decrease in attraction towards isoamyl alcohol than *dop-*2*(vs105)* and *gpa-14(pk347)* (p = 0.35 for naive and p < 0.05 for conditioned animals, ANOVA). Additionally, the conditioned response of the *gpa-14;dop-*2 double mutant is the same as either single mutant (p = 0.46, One-Way ANOVA). (**B**) When conditioned in the presence of exogenous dopamine, no statistical significance was noted between the wild types and the mutants (p = 0.65 for naives and p = 0.88 for conditioned, One Way ANOVA). (**C**) As a control, 3-day-old synchronized worms were exposed to 3 μl of isoamyl alcohol in the presence of *E. coli* for 90 minutes and tested for their attraction to 1:100 isoamyl alcohol. No significant difference in attraction was observed for wild type or either of the mutants [*dop-2(vs105)* and *gpa-14(pk347)*] before or after treatment (p = 0.85 for naives and p = 0.89, One Way ANOVA). Bars represent SEM values n ≥ 3 assays for each strain.

It has been previously reported that *dop-2(vs105)* mutants are deficient in chemotaxis based associative learning paradigms [9]. We tested the performance of *gpa-14(pk347)* in a learning assay in which the chemo-attractant isoamyl alcohol (IAA) was paired with starvation [9]. For conditioning, the animals were exposed to 3 μl of IAA for 90 minutes. IAA (2μl) diluted to 1/100 in ethanol was applied to the gradient spot and ethanol (2μl) was applied to the diluent point, and worms were placed equidistant to the two points. Plates were left undisturbed for one hour, after which the animals were counted to calculate a chemotaxis index for each plate [9,24]. Naive worms showed strong attraction towards to 1:100 dilution of IAA. After conditioning, N2 worms displayed significantly reduced attraction to isoamyl alcohol compared to both *gpa-14(pk347)* and *dop-2(vs105)* (Figure 3A). Additionally, there was no significant difference between the learning capacity of the *gpa-14(pk347)* or the *dop-2(vs105)* strains. Similar associative learning results were obtained using paradigms that used non-volatile sodium chloride as unconditioned stimulus (Additional file 2). In control experiments we did not observe any decrease in attraction to isoamyl alcohol when the animals had been previously exposed to isoamyl alcohol in the presence of food/*E. coli*, confirming that the decrease in response observed upon conditioning in the absence of *E. coli* is a learned response and not due to adaptation [Figure 3C].

In order to test whether DOP-2 and GPA-14 exert their influence on the observed adaptive behavior through the same pathway, a *gpa-14(pk347);dop-2(vs105)* double mutant was generated. This double mutant adapted significantly faster than N2 at rates similar to those observed for *gpa-14(pk347)* or *dop-2(vs105)* single mutants (Figure 2A). No cumulative abnormalities were observed in the double mutant indicating a genetic interaction between *dop-2* and *gpa-14*.

### Dopamine supplements revert behavioral deficits in mutants

It has been shown that dopamine deficiency results in faster habituation rates while abundance in dopamine has been correlated with slower habituation rates [16,17]. Behavioral deficits attributed to low levels of dopamine have been ameliorated by providing exogenous dopamine [8,15,16]. Upon assaying in the presence of 5mM exogenous dopamine, we were able to bring habituation rates of both the single-mutants [*gpa-14(pk347) and dop-2(vs105)*] as well as the double mutant [*gpa-14(pk347);dop-2(vs105)*] to levels similar to the habituation rate of wild type N2 animals (Figure 2B). Addition of exogenous dopamine was also able to rescue the conditioned associative learning deficits for the *gpa-14(pk347)* and *dop-2(vs105)* deletions as well as the *gpa-14(pk347);dop-2(vs105)* double deletion mutants (Figure 3B).

## Discussion

We provide genetic and phenotypic support to our previously reported *in vitro* interaction between the DOP-2 dopamine receptor and the GPA-14 Gα_i_ subunit [21]. The *gpa-14(pk347)* and *dop-2(vs105)* mutants display faster rates of habituation and diminished associative learning capacities. Faster mechanosensitive habituation has been reported for mutants in *dop-1*, which codes for a D1-like receptor [15], and crosstalk between AVM touch neurons (which express a D1-like receptor, DOP-1) and ADE dopaminergic neurons (which express the D2-like DOP-2 receptor) has been proposed previously [16]. It will be interesting to explore whether DOP-1 and DOP-2 work as an antagonistically coordinating pair as indicated by the pharmacological properties of D1- and D2- receptor types [5]. Supplementary exogenous dopamine in either of the single mutant or the double mutant used in this study reverted the observed phenotypic abnormalities, indicating that DOP-2 and GPA-14 play an upstream role in regulating the dopaminergic pathway of behavioral plasticity in worms. In addition to its role in learning and memory, dopamine has been reported to regulate food encounter response of *C. elegans*[2]. According to our results, the deletion of *dop-2* or *gpa-14* has no effect on their food encounter response. This could imply that, although dopamine activity is reduced in these mutants, its reduction may not be significant enough to affect their food encounter response. Another possibility is that basal slowing may not be as sensitive to dopamine levels as mechanosensation and chemosensensation.

Absence of any additional or cumulative abnormalities in the *gpa-14(pk347);dop-2(vs105)* double mutant provides genetic support towards an interaction between *dop-2* and *gpa-14* and that their protein products participate in the same molecular pathway during behavior plasticity. Apparently, the observed habituation and associative learning behaviors in both mutants [*gpa-14(pk347) and dop-2(vs105)*] may give an impression of contradictory phenotypes. While diminished associative learning clearly correlates with poorer neuronal summation, faster habituation does not necessarily draw a parallel with an antagonistic explanation. On the other hand faster habituation may correlate with ignoring a cue earlier than optimum assuming that WT habituation represents an optimal rate. In summary, examining the behavioral profile of *dop-2(vs105)* and *gpa-14(pk347)* deletion mutants has revealed that both show similar aberrations in plasticity, and our results with the *dop-2;gpa-14* double mutant indicate that GPA-14 and DOP-2 work together in the same pathway. In order to rule out the contribution of background mutations, follow-up experiments using RNAi knockdowns or transgenic rescue of the mutants can provide required validation of the results presented here.

These results are significant in that neuroimaging studies in humans indicate that deficient feedback monitoring in the cortex is associated with learning deficits due to D2 receptor polymorphisms [25,26]. The ASIC and DAT-1 pathways are also known to modulate dopamine release in the dopaminergic neurons including ADEs and have been proposed to work in conjunction with DOP-2 ([8] and Additional file 3). However, the nature of the upstream or parallel role of DOP-2 in the plasticity pathway is not understood at the molecular level. It is conceivable that DOP-2 may act as an auto-receptor in the ADE neurons and upon stimulation it transduces the extracellular dopamine signal through the GPA-14 Gα_i_ subunit [8,21]. Alternately, DOP-2 may exert its influence at the somatodendritic membrane, given the dual localization of mammalian D2 receptors at both cellular compartments of dopaminergic neurons [27]. Further investigations are needed and it will be interesting to identify downstream components of the ASIC, DAT-1 and DOP-2 pathways and their crosstalk in modulating precise levels of neurotransmitter in the synaptic cleft.

## Abbreviations

gpa-14: Gene for G-protein alpha subunit-14; GPA-14: Protein product coded by *gpa-14*; dop-2: Gene for D2 like dopamine receptor; DOP-2: *dop-2* protein product; ASIC: Acid sensing ion channel; asic-1: Gene for a subunit of *C. elegans* ASIC.

## Competing interests

The authors declare that they have no competing interests.

## Authors’ contributions

Conceived project: NT, SH. Designed experiments: MM, RF, PP, SH. Performed experiments: MM, RF, RM. Wrote manuscript: NT, SH. All authors have read and approve the final manuscript.

## Supplementary Material

Additional file 1 **Basal slowing rates for *dop-2(vs105) *and *gpa-14(pk347) *mutants do not differ from wild type animals.** Body bends of 3 day old individual worms were counted for 20 seconds. Basal slowing assay with food is represented by the white bars while basal slowing assay without food is represented by the grey bars. Each bar indicates the average body bends/20 seconds in three experiments. Error bars indicate SEM (n = 60 for each strain; P = 0.067 without food and P = 0.178 with food). Click here for file

Additional file 2 ***gpa-14(pk347) *deletion mutants displayed associative learning deficits when paired with either soluble or volatile chemicals, isoamyl alcohol or sodium chloride, respectively.** n = 90 in three experiments; *: P < 0.001; ns: non-significant; unpaired t-test. Click here for file

Additional file 3 **A model for the molecular interactions modulating neurotransmitter levels at a *C. elegans *dopaminergic synapse {modified from **[8]**}.** Release of dopamine can activate auto-receptor function of DOP-2 to initiate signal transduction through GPA-14 ([21], and this report). In parallel, the accompanying drop in synaptic pH due to the release of H^+^ ions from the acidified vesicles activates acid sensing cation channels (ASIC). Stimulation of DOP-2 and activation of ASIC call allow two molecular loops in the presynaptic neuron so as to modulate levels of dopamine in the synaptic cleft. These two pathways are likely to crosstalk through relay molecule/s that remain unknown as yet. Click here for file

## References

[B1] Kandel ER The molecular biology of memory storage: a dialog between genes and synapses Biosci Rep 2001 21 5 565 611 10.1023/A:1014775008533 12168768

[B2] Sawin ER Ranganathan R Horvitz HR *C. elegans* locomotory rate is modulated by the environment through a dopaminergic pathway and by experience through a serotonergic pathway Neuron 2000 26 619 631 10.1016/S0896-6273(00)81199-X 10896158

[B3] Khan ZU Muly EC Molecular mechanisms of working memory Behav Brain Res 2011 219 2 329 341 10.1016/j.bbr.2010.12.039 21232555

[B4] Wei-Dong Y Spealman RD Zhang J Dopaminergic signaling in dendritic spines Biochem Pharmacol 2009 75 11 2055 2069 10.1016/j.bcp.2008.01.018 PMC2443745 18353279

[B5] Beaulieu JM Gainetdinov RR The physiology, signaling, and pharmacology of dopamine receptors Pharmacol Rev 2011 63 182 217 10.1124/pr.110.002642 21303898

[B6] Williams JM Galli A The dopamine transporter: a vigilant border control for psychostimulant action Handb Exp Pharmacol 2006 175 215 232 10.1007/3-540-29784-7_11 16722238

[B7] Pidoplichko VI Dani JA Acid-sensitive ionic channels in midbrain dopamine neurons are sensitive to ammonium, which may contribute to hyperammonemia damage Proc Natl Acad Sci USA 2006 103 30 11376 11380 10.1073/pnas.0600768103 16847263 PMC1544094

[B8] Voglis G Tavernarakis N A synaptic DEGENaC ion channel mediates learning in *C. elegans* by facilitating dopamine signaling EMBO J 2008 27 3288 3299 10.1038/emboj.2008.252 19037257 PMC2609744

[B9] L’hirondel M Cheramy A Godeheu G Artaud F Saiardi A Borrelli E Glowinski J Lack of autoreceptor-mediated inhibitory control of dopamine release in striatal synaptosomes of D2 receptor-deficient mice Brain Res 1998 792 253 262 10.1016/S0006-8993(98)00146-2 9593923

[B10] Nuttley W Harbinder S van der Kooy D Genetic dissection and kinetics of opposing attractive and aversive components triggered in response to benzaldehyde in *C. elegans* Learn Mem 2001 8 3 170 181 10.1101/lm.36501 11390637 PMC311371

[B11] Saeki S Yamamoto M Iino Y Plasticity of chemotaxis revealed by paired presentation of a chemoattractant and starvation in the nematode *Caenorhabditis elegans* J Exp Biol 2001 204 10 1757 1764 11316496 10.1242/jeb.204.10.1757

[B12] Ardiel EL Rankin CH An elegant mind: Learning and memory in *Caenorhabditis elegans* Learn Memory 2010 17 191 201 10.1101/lm.960510 20335372

[B13] White JG Southgate E Thomson JN Brenner S The structure of the nervous system in the nematode *Caenorhabditis elegans* Philos Trans R Soc Lond B Biol Sci 1986 314 1 340 10.1098/rstb.1986.0056 22462104

[B14] Suo S Sasagawa N Ishiura S Cloning and characterization of a *Caenorhabditis elegans* D2-like dopamine receptor J Neurochem 2003 86 869 878 10.1046/j.1471-4159.2003.01896.x 12887685

[B15] Sanyal S Wintle RF Kindt KS Nuttley WM Arvan R Fitzmaurice P Bigras E Merz DC Hébert TW van der Kooy D Schafer WR Culotti JG Van Tol HH Dopamine modulates the plasticity of mechanosensory responses in *Caenorhabditis elegans* EMBO J 2004 23 473 482 10.1038/sj.emboj.7600057 14739932 PMC1271763

[B16] Kindt KS Quast KB Giles AC De S Hendrey D Nicastro I Rankin CH Schafer WR Dopamine mediates context-dependent modulation of sensory plasticity in *C. elegans* Neuron 2007 55 4 662 676 10.1016/j.neuron.2007.07.023 17698017

[B17] Ezak MJ Ferkey DM The *C. elegans* D2-like dopamine receptor DOP-3 decreases behavioral sensitivity to the olfactory stimulus 1-octanol PLoS One 2010 5 3 e9487 10.1371/journal.pone.0009487 20209143 PMC2830454

[B18] Ezcurra M Tanizawa Y Swaboda P Schafer WR Food sensitizes *C. elegans* avoidance behaviours through acute dopamine signaling EMBO J 2011 30 6 1110 1122 10.1038/emboj.2011.22 21304491 PMC3061029

[B19] Bastiani C Mendel J Heterotrimeric G proteins in *C. elegans*. WormBook, ed. The *C. elegans* Research Community WormBook 2006 http://www.wormbook.org 10.1895/wormbook.1.75.1 PMC4781550 18050432

[B20] Jansen G Thijssen KL Werner P Van der Horst M Hazendonk E Plasterk RH The complete family of genes encoding G-proteins of *Caenorhabditis elegans* Nat Genet 1999 21 414 419 10.1038/7753 10192394

[B21] Pandey P Harbinder S The *Caenorhabditis elegans* D2-like dopamine receptor DOP-2 physically interacts with GPA-14, a Gαi subunit J Mol Signal 2012 7 1 3 10.1186/1750-2187-7-3 22280843 PMC3297496

[B22] Rose JK Rankin CH Analysis of Habituation in *Caenorhabditis elegans* Learn Mem 2001 8 63 69 10.1101/lm.37801 11274251

[B23] Giles AC Rankin CH Behavioral and genetic characterization of habituation using *Caenorhabditis elegans* Neurobiol Learn Mem 2009 92 2 139 146 10.1016/j.nlm.2008.08.004 18771741

[B24] Bargmann CI Horvitz HR Chemosensory neurons with overlapping functions direct chemotaxis to multiple chemicals in *Caenorhabditis elegans* Neuron 1991 7 5 729 742 10.1016/0896-6273(91)90276-6 1660283

[B25] Klein TA Neumann J Reuter M Hennig J von Cramon DY Ullsperger M Genetically determined differences in learning from errors Science 2007 2007 318 1642 1645 18063800 10.1126/science.1145044

[B26] Jocham G Klein TA Neumann J Cramon DY Reuter M Ullsperger M Dopamine DRD2 polymorphism alters reversal learning and associated neural activity J Neurosci 2009 29 12 3695 3704 10.1523/JNEUROSCI.5195-08.2009 19321766 PMC2694507

[B27] Jomphe C Tiberi M Trudeau LE Expression of D2 receptor isoforms in cultured neurons reveals equipotent autoreceptor function Neuropharmacol 2006 50 5 595 605 10.1016/j.neuropharm.2005.11.010 16412480

