# Supplementary figures and images for: GPA-14, a Gαi subunit mediates dopaminergic behavioral plasticity in C. elegans
Source: Behav Brain Funct. 2013 Apr 22;9:16. doi: 10.1186/1744-9081-9-16 (PMC3679979; doi:10.1186/1744-9081-9-16)

## Slide 1
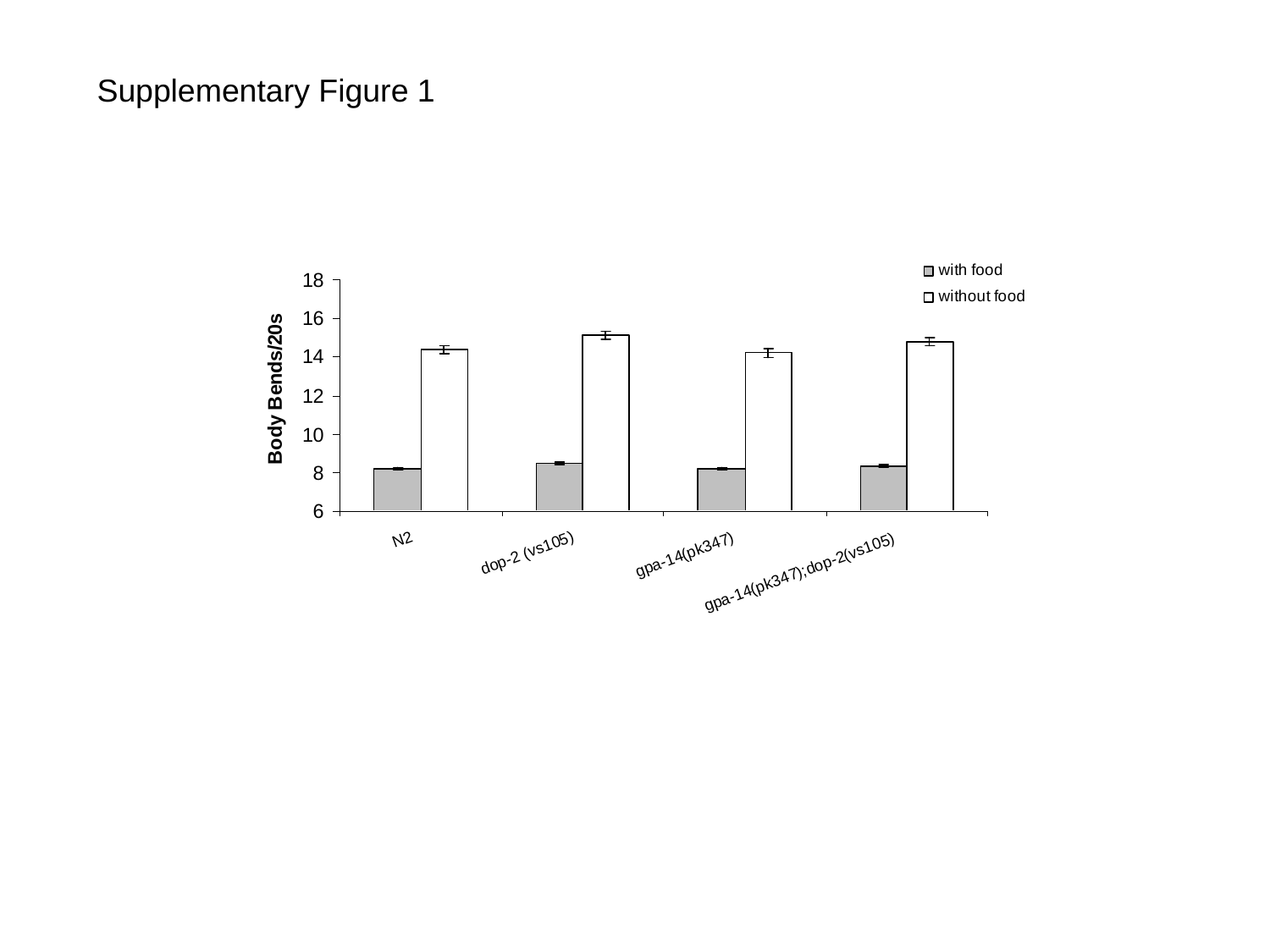

Supplementary Figure 1

Supplement: Additional file 1 — Basal slowing rates for dop-2(vs105) and gpa-14(pk347) mutants do not differ from wild type animals. Body bends of 3 day old individual worms were counted for 20 seconds. Basal slowing assay with food is represented by the white bars while basal slowing assay without food is represented by the grey bars. Each bar indicates the average body bends/20 seconds in three experiments. Error bars indicate SEM (n = 60 for each strain; P = 0.067 without food and P = 0.178 with food). [file 1744-9081-9-16-S1.ppt]

## Slide 1
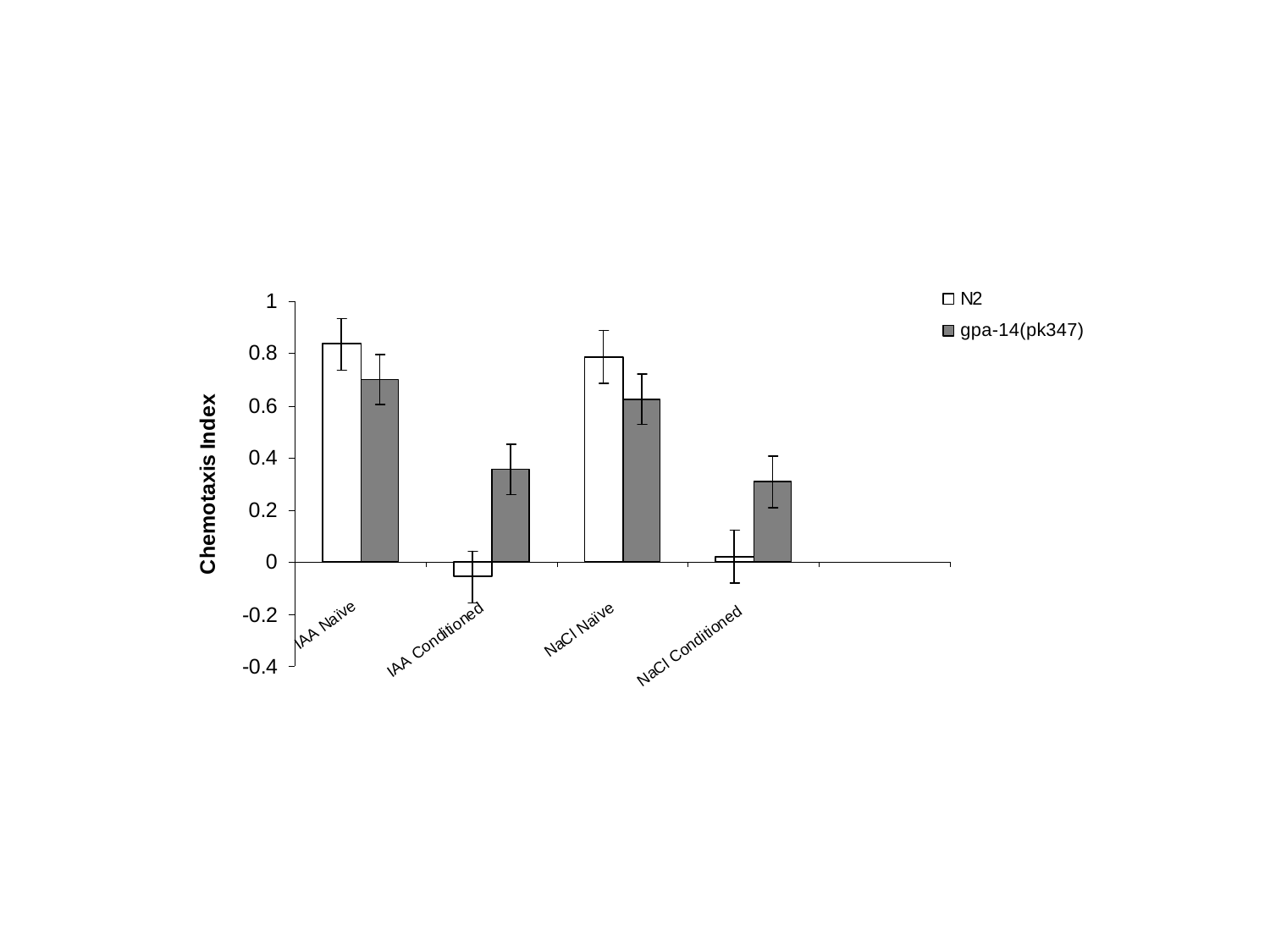

Supplement: Additional file 2 — gpa-14(pk347) deletion mutants displayed associative learning deficits when paired with either soluble or volatile chemicals, isoamyl alcohol or sodium chloride, respectively. n = 90 in three experiments; *: P < 0.001; ns: non-significant; unpaired t-test. [file 1744-9081-9-16-S2.ppt]

## Slide 1
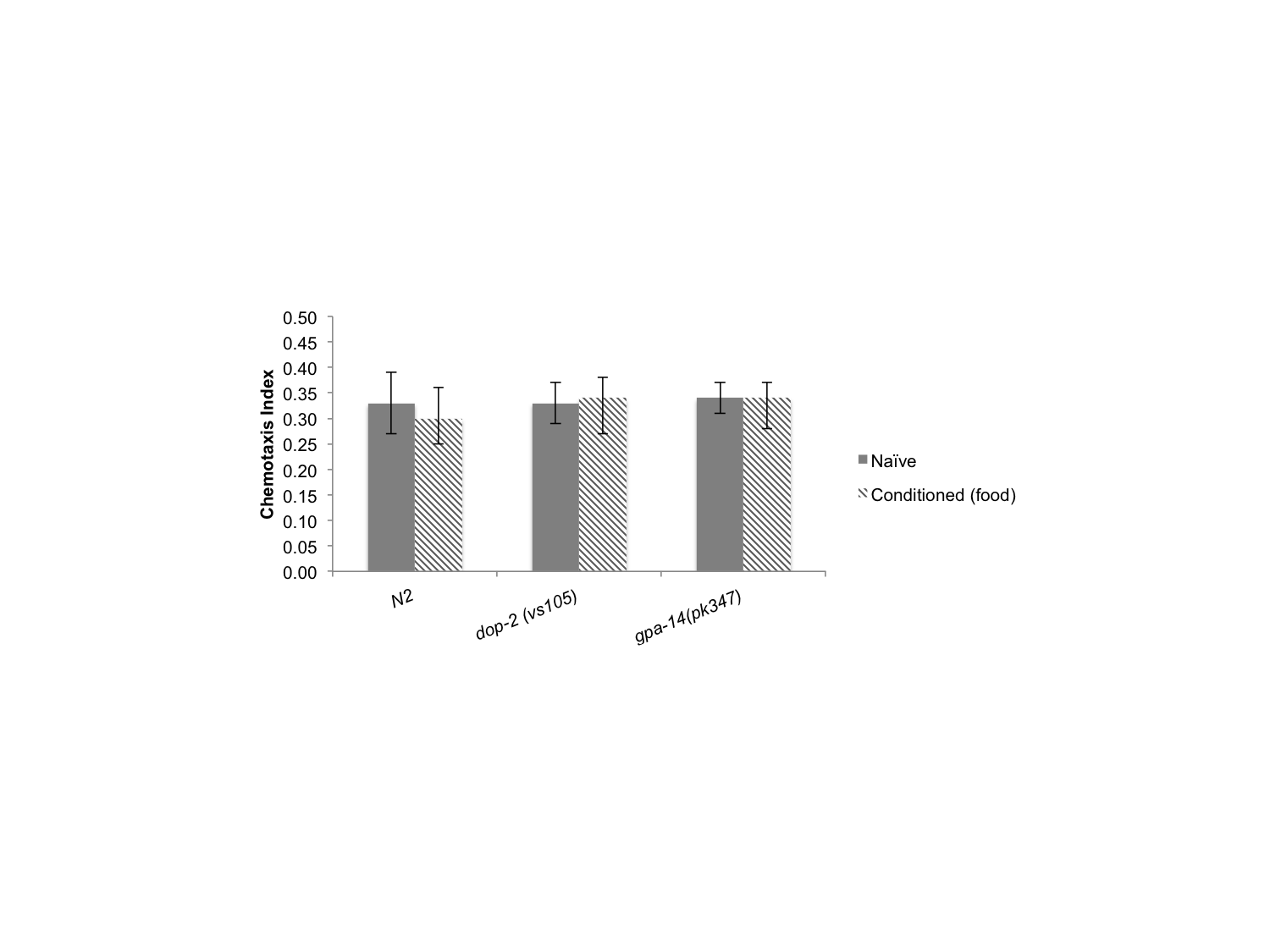

Supplement: Additional file 3 — A model for the molecular interactions modulating neurotransmitter levels at a C. elegans dopaminergic synapse {modified from [8]}. Release of dopamine can activate auto-receptor function of DOP-2 to initiate signal transduction through GPA-14 ([21], and this report). In parallel, the accompanying drop in synaptic pH due to the release of H+ ions from the acidified vesicles activates acid sensing cation channels (ASIC). Stimulation of DOP-2 and activation of ASIC call allow two molecular loops in the presynaptic neuron so as to modulate levels of dopamine in the synaptic cleft. These two pathways are likely to crosstalk through relay molecule/s that remain unknown as yet. [file 1744-9081-9-16-S3.ppt]
